# Multi‐Omics Analyses Elucidate the Venom Components of the Wasp *Vespa mandarinia*

**DOI:** 10.1002/ece3.73724

**Published:** 2026-05-27

**Authors:** Yuan‐Chong Shi, Hai‐Feng Mo, Shu‐Hui Yu, Xiao‐Yun Niu, Pu Yang

**Affiliations:** ^1^ Institute of Highland Forest Science Chinese Academy of Forestry Kunming China; ^2^ College of Agriculture and Life Sciences Kunming University Kunming China; ^3^ Key Laboratory of Breeding and Utilization of Resource Insects Of National Forestry and Grassland Administration Kunming China; ^4^ Yunnan Key Laboratory of Breeding and Utilization of Resource Insects Kunming China

**Keywords:** hyaluronidase, metabolome, peptidome, proteome, venom, venom dipeptidyl peptidase 4, wasp

## Abstract

The wasp *Vespa mandarinia* was widely reared in Yunnan province in China. It is characterized by notable toxicity, large body size, and strong invasiveness. Its extremely potent venom and high output lead to aggressive behavior. To elucidate the composition of *V. mandarinia* venom, proteome, peptidome, and metabolome analyses were conducted in this study. Proteome analysis identified 2189 proteins, among which 842 were proteases, and 20 were venom‐related proteases. 1294 proteins were identified as nonenzymatic proteins, with 2 toxic proteins. Moreover, hyaluronidase, venom dipeptidyl peptidase 4, and phospholipase A1 were detected. Peptidome analysis detected 1263 peptides, 70 of which were proteolytic peptides, including one phospholipase A2 isozyme. Among the 223 nonenzymatic peptides, two were characterized as vespid chemotactic peptide and the mastoparan‐like peptide. Metabolome analysis tentatively annotated 918 compounds in positive mode versus 499 in negative mode. Organic heterocyclic compounds, organic acids, and their derivatives were the most abundant superclasses of venom metabolites. γ‐aminobutyric acid, N‐acetylhistamine, tryptamine, and dopamine were also identified. These results uncovered key toxic components, including venom‐related proteases, toxic peptides, and bioactive metabolites. It constituted a comprehensive molecular basis for understanding toxicity and biological functions of *V. mandarinia* venom.

## Graphical abstract

The wasp *Vespa mandarinia* was widely reared in Yunnan province. The venom was responsible for their aggressive behavior. Tweety proteases, hyaluronidase, venom dipeptidyl peptidase 4, and phospholipase A1 and A2 isozyme were identified. In addition, organic heterocyclic compounds, organic acids, and derivatives were found. These components were important to the toxicity of *V. mandarinia*.

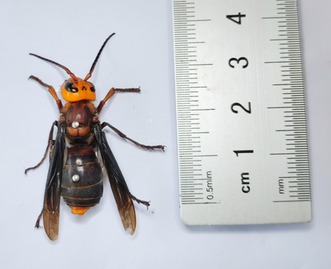

## 1 Introduction

**FIGURE 1:**
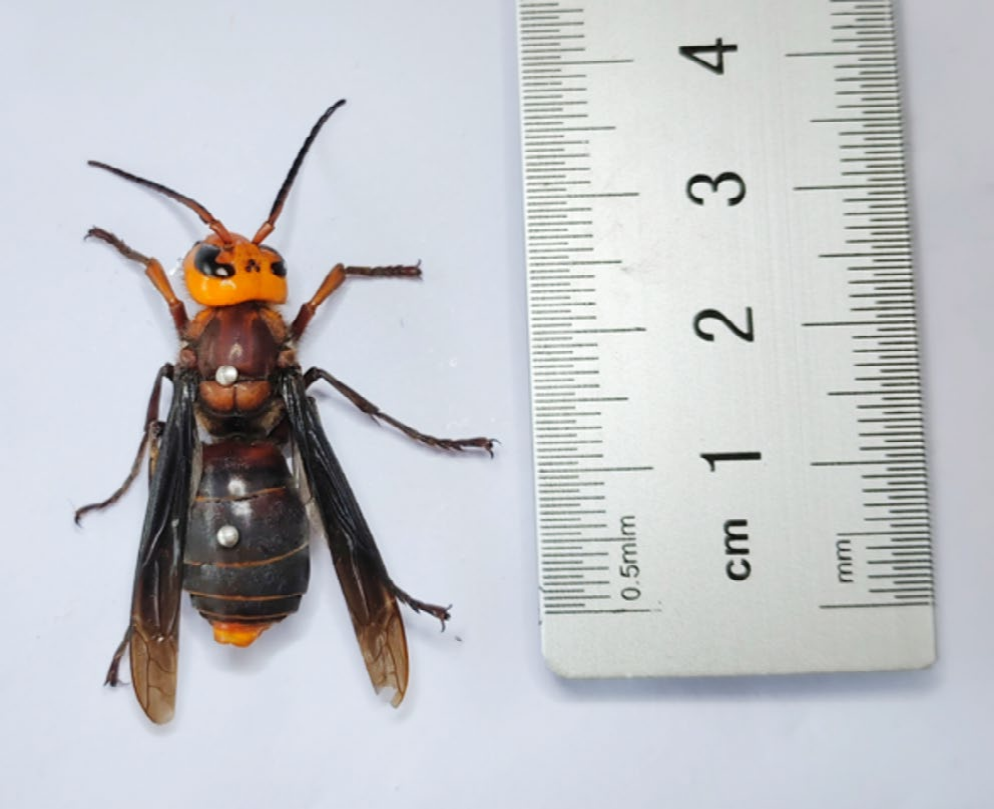
A female worker wasp of *Vespa mandarinia* collected from Yunnan Province, China.

Figure 1 shows a female *Vespa mandarinia* collected in Yunnan Province, China.

*Vespa mandarinia* is a eusocial insect belonging to the family Vespidae (Hymenoptera). The adult body length ranges from 30 to 50 mm. The head is orange—yellow, the thorax is black, and the abdomen has alternating yellow and black stripes. The stinger of the female wasp is approximately 6 mm long (Dong et al. 2022). *V. mandarinia* is native to Asia and mainly distributed in regions including China, Japan, Vietnam, and India (Matsuura and Sakagami 1973; Nuñez‐Penichet et al. 2021). It was an invasive species in many countries (Zhu et al. 2020).

*Vespa mandarinia* is renowned as one of the most ferocious representatives of wasps (Matsuura and Sakagami 1973). It was regarded as a top‐level insect predator. The diet of *V. mandarini* encompasses beetles, spiders, other hymenopteran insects, and even other *Vespa* insects, along with tree sap, nectar, and certain fruits (Matsuura and Sakagami 1973; Spears 2020; Topitzhofer et al. 2022). The components of the venom typically cause pain and allergic reaction. In some cases, repeated stings can even lead to death (Herrera et al. 2020; Vetter et al. 1999).

*Vespa mandarinia* cultivated in Yunnan Province displays a distinct morphological characteristic by a large body size, which is associated with high toxicity and a restricted distribution range. The body size is large, usually more than 3 cm in length (Figure 1). They were reared to obtain pupae, which is an expensive food (Ghosh et al. 2021; Kiewhuo et al. 2022; Ying et al. 2001). Their venom had significant medicinal value because of the numerous biochemical components (Abd El‐Wahed et al. 2021). Research has demonstrated that *V. mandarinia* not only has extremely potent toxicity but also exhibits a high venom output. It has good antibacterial effects and cytotoxicity (Smeding et al. 2020).

The venom of social wasps contains peptidic toxins, allergens, and amines like 5‐hydroxytryptamine (Dos Santos‐Pinto et al. 2018; Habermann 1972; Herrera et al. 2020). Venom components in certain wasp species have been identified, such as *Vespa bicolor* (Wu et al. 2022). Their study identified three proteins and two abundant bioactive substances with confirmed allergenic activity (Wu et al. 2022). Yoon conducted a transcriptomic analysis of aculeate bumblebees and wasps (Yoon et al. 2020). *V. mandarinia* is one of the most poisonous *Vespa*. The lethal capacity of four vespid species from Asia has been compared, and *V. mandarinia* has the highest venom content, with values of 4.1 μL and 1.10 mg dry weight per worker (Schmidt et al. 1986).

The culture scale of *V. mandarinia* was large in Yunnan Province. Some local residents raise *V. mandarinia* to sell its pupa for money and collect the venom or use adults to produce medicinal liquor. In recent years, it has been exported to areas around Yunnan Province. *V. mandarinia* usually establishes colonies and expands the population in imported areas in a short period of time. When humans disrupt *V. mandarinia* or encroach upon their nests, these wasps initiate a collective counterattack. Predictably, the ecological role and potential diffusion of *V. mandarinia* is appealing.

In this study, proteome, peptidome, and metabolome analyses were employed to comprehensively analyze the venom components of *V. mandarinia*. The results of these analyses are expected to precisely identify the various venom components of *V. mandarinia* and provide information for understanding the pronounced aggression of *V. mandarinia* in Yunnan Province.

## 2 Methods

### 2.1 Proteomics Analyses

The posterior abdominal region of female *V. mandarinia* individuals was detached from the body using forceps, and the surrounding abdominal wall was carefully removed. The venom sac was found. The venom sacs from female wasps were detached and placed in freezing tubes in liquid nitrogen immediately. The venom sacs of female wasps were detached and placed in freezing tubes in liquid nitrogen immediately. About 25 mg of venom sac samples were used. 50 mM ammonium bicarbonate was mixed with venom sacs and centrifuged for 15 min with a centrifugal force of 25,000 × g after cutting tissues. The tissue precipitation was removed. To precipitate total proteins, prechilled acetone was added to the supernatant; after 2 h incubation at −20°C, the protein mixture was subjected to centrifugation at 25,000 × g (4°C, 15 min), and the supernatant was removed. The air‐dried precipitate was mixed with 50 mM ammonium bicarbonate. An ultrasonic apparatus was used to promote the dissolution of protein. Then it was mixed with dithiothreitol (DTT, 10 mM) and incubated for 30 min (37°C). The mixture was treated with 55 mM iodoacetamide, and then incubated for 45 min in a dark environment. The protein concentration was detected via the Bradford method.

Protein was first separated by sodium dodecyl sulfate–polyacrylamide gel electrophoresis (SDS–PAGE). The sample was subsequently cut into 1 mm gel fragments. After destaining, dehydration, sulfhydryl groups reduction, and blocking, trypsin was added to the gel fragments. Five volumes of 50% acetonitrile (ACN) were added to the digested gel fragments, and after vibration for 5 min, the mixture was centrifuged (5000 × g, 1 min). Five volumes of 100% ACN were mixed with the supernatant. After shaking for 5 min, the sample was centrifuged again. The new supernatant was transferred in another tube, and centrifuged for 5 min. Finally, the separated supernatant was collected and freeze‐dried.

The dried proteins were redissolved in mobile phase A containing 2% ACN and 0.1% formic acid. After centrifugation (20,000 × g, 10 min), the supernatant was injected into an Eksigent ultra2D (SCIEX, USA) to initiate the liquid phase separation program.

High‐performance liquid chromatography (HPLC) with a TripleTOF 5600 mass spectrometer (SCIEX, USA) was used for protein analysis. In this study, a Nanospray III source (SCIEX, USA) was used as the ion source, and a quartzose needle (New Objectives, USA) was used as the emitter. Raw data were converted into mass peak data. The acquired data were subsequently searched against a customized protein database derived using Mascot (v 2.3.02). This custom database was constructed based on the assembled genome and its Maker‐predicted gene models. The results were subjected to quality control using Percolator.

Functional enrichment analysis was performed using the hypergeometric test for statistical evaluation. The background set consisted of all protein‐coding genes annotated in the *V. mandarinia* genome.

The amino acid sequences of hyaluronidase, venom dipeptidyl peptidase 4, phospholipase A1, and venom serine protease precursor were downloaded from the NCBI GenBank database. For each target gene, amino acid sequences from multiple related species were aligned by using ClustalW (v2.1). The alignment result was manually inspected and trimmed to ensure data quality. The aligned sequences were imported into MEGA 11 software. The phylogenetic trees were generated by the Neighbor‐Joining (NJ) method, and the robustness was evaluated by bootstrap analysis with 1000 replicates.

### 2.2 Peptidome Analyses

Venom samples used for peptidome analyses were prepared in the same procedure as those for proteome analyses except that the trypsin digestion step was omitted. The 10 kDa filter membrane was used for filtering. The solid‐phase extraction (SPE) C18 cartridge was subsequently used to purify the polypeptide extract. MaxQuant (Arien et al. 2018) 2.0.1.0 (http://www.maxquant.org) was used for peak recognition, information extraction, and sequence identification with the raw data from the Orbitrap.

### 2.3 Metabolome Analyses

Venom samples used for metabolome analyses were similar to that used for proteome analyses. A total of eight hundred microliters of extraction solution and the two internal standards were added to the tube. The sample was then ground for 5 min at 50 Hz using a JXFSTPRP tissue grinder (Jing Xin, China) and two steel balls. Then it was broken by ultrasonic disruption.

The solutions were centrifuged (25,000 × g, 4°C, 15 min). Then, approximately six hundred microliters of the supernatant were extracted and placed into a freezing vacuum concentrator (Maxi Vacbeta, Gene Company). After drying, the methanol solution was used to redissolve samples. The mixture was vibrated for 1 min, then subjected to an ultrasonic bath at 4°C for 10 min. The solutions were extracted via centrifugation. Quality control (QC) was performed. Repeatability and stability of LC–MS were analyzed.

The metabolites were analyzed by chromatography. An ACQUITY UPLC BEH C18 column (1.7 μm 2.1 * 100 mm, Waters, USA) was utilized for separation, with elution being performed.

A Q Exactive mass spectrometer (Thermo Fisher Scientific, United States) was utilized to obtain first‐order and second‐order mass spectrometry data. To ensure the reliability of data, the injection order of samples was randomized, and a QC sample was inserted after every ten experimental samples.

Raw LC–MS/MS data were analyzed using Compound Discoverer 3.1 (Thermo Fisher Scientific, USA). Peak extraction, ion combination, missing value imputation, retention time correction (both intra‐ and intergroup), background peak filtering, and metabolite identification were performed. The final dataset was generated, containing peak area, compound detection, identification results, and retention time. Metabolite identification was performed based on the BGI Library.

## 3 Results

### 3.1 Proteomic

A total of 62,689 spectra, 17,100 peptides, and 2189 proteins were identified. Approximately one‐fifth of the proteins had 5–10 unique spectra. The protein molecular weight distribution data revealed that the protein mixture was composed of different 10–100 kDa molecular weight proteins. Moreover, the richest proteins were distributed at 20–30 kDa, and more than half of the proteins were distributed at 10–60 kDa.

According to the functional annotation from the Swiss‐Prot database, 2136 proteins (97.6%) were annotated (Table S1). Moreover, 25 proteins were identified via the iVenomDB database, and 672 proteins were identified via the T3DB database (Tables S2 and S3).

**FIGURE 2:**
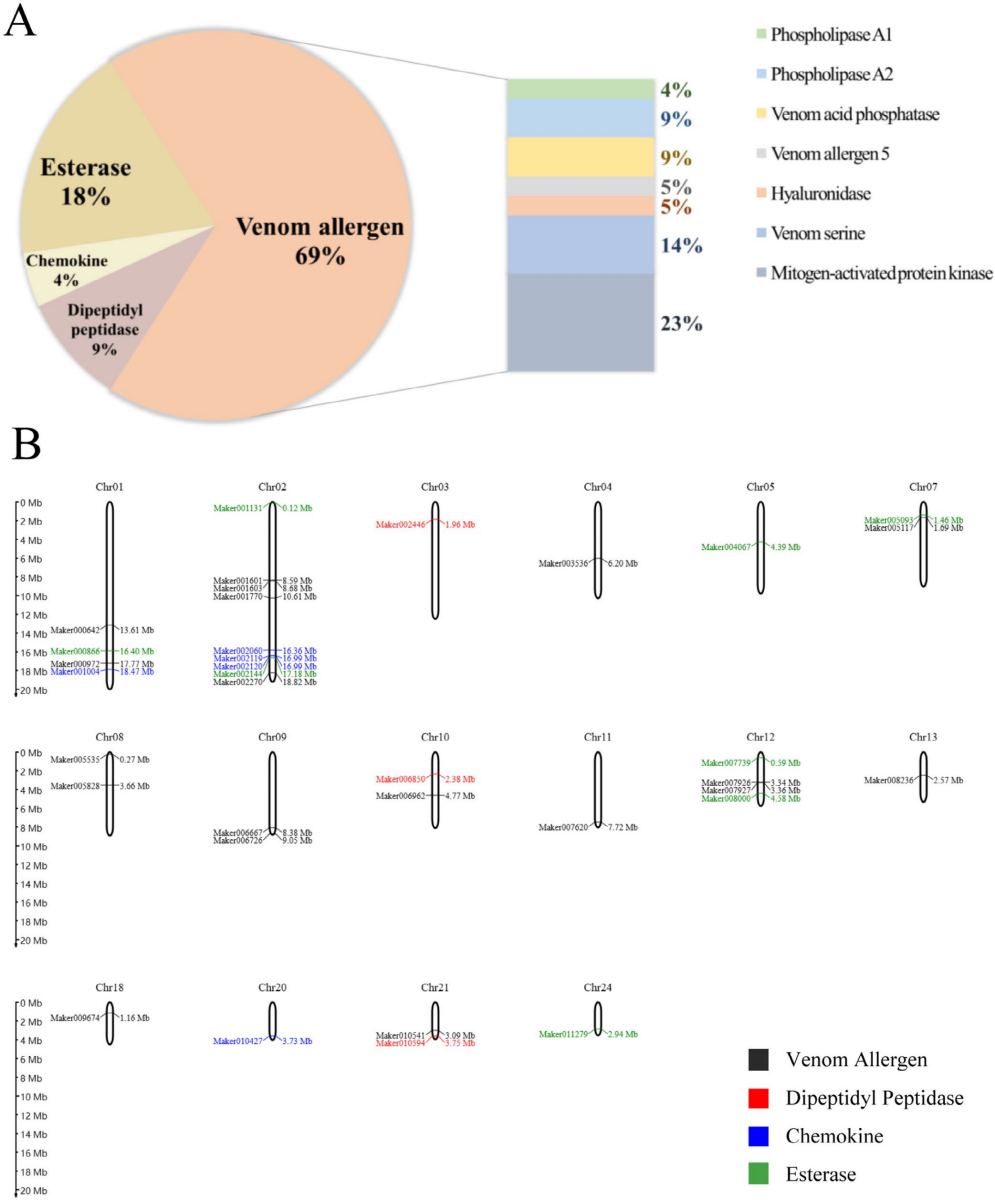
The classification and chromosome location of some venom genes. (A) Classification of 22 venom proteins on the basis of the SwissProt database of *V. mandarinia* venom: Venom allergens accounted for 69% of the venom proteins, including 4% phospholipase A1, 9% phospholipase A2, 9% venom acid phosphatase, 5% venom allergen 5%, 5% hyaluronidase, 14% venom serine, and 23% mitogen‐activated protein kinase. Esterases account for 18%, chemokines account for 4%, and dipeptidyl peptidases account for 9%. (B) Gene localization in *the V. mandarinia* chromosomes of venom proteins: The black genes represent venom allergens, the red genes represent dipeptidyl peptidases, the blue genes represent chemokines, and the green genes represent esterases.

**FIGURE 3:**
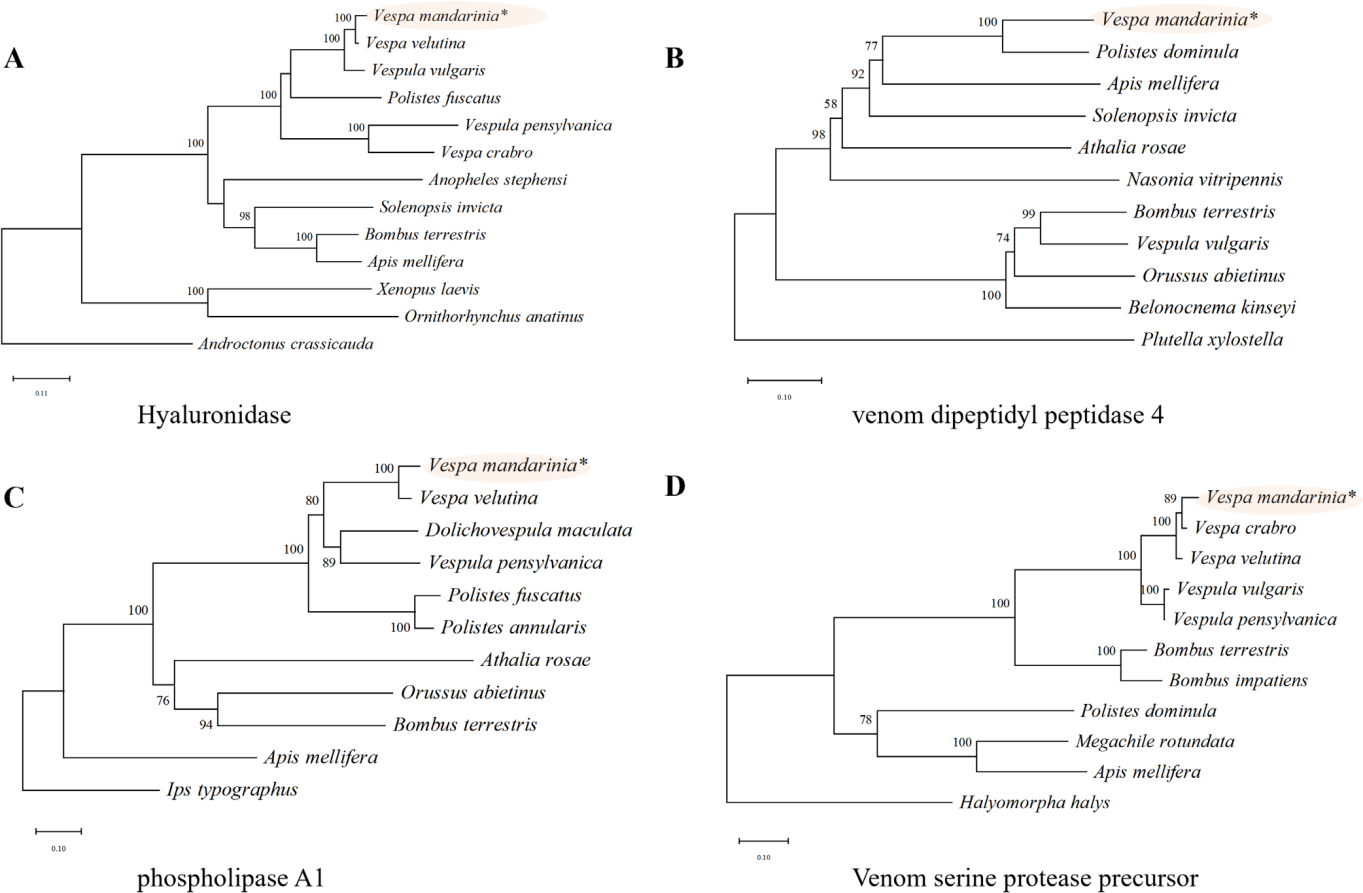
Evolutionary tree of four genes related to *V. mandarinia* toxicity. (A) Hyaluronidase. *Apis mellifera* : NP_001011619.1; *Xenopus laevis* : NP_001165445.1; *Solenopsis invicta* : XP_011161185.1; *Bombus terrestris* : XP_048268069.1; *Androctonus crassicauda*: UCR74866.1; *Vespula vulgaris*: XP_050858958.1; *Vespula pensylvanica* : XP_043676905.1; *Vespa velutina*: XP_047355440.1; *Vespa crabro* : XP_046814697.1; *Polistes fuscatus* : XP_043505678.1; *Ornithorhynchus anatinus* : XP_001507937.2; *Anopheles stephensi*: XP_035901645.1. (B) Venom dipeptidyl peptidase 4. *Apis mellifera* : XP_026299326.1; *Belonocnema kinseyi*: XP_033227070.1; *Bombus terrestris* : XP_048264207.1; *Nasonia vitripennis*: XP_032455093.1; *Vespula vulgaris*: XP_050854187.1; *Athalia rosae*: XP_012252193.2; *Orussus abietinus*: XP_023289115.1; *Plutella xylostella*: XP_037977148.2; *Polistes dominula*: XP_015174446.1; *Solenopsis invicta* : XP_025992386.1. (C) Phospholipase A1. *Athalia rosae*: XP_012251606.2; *Dolichovespula maculata* : CAA47341.1; *Apis mellifera* : XP_394360.2; *Bombus terrestris* : XP_012174894.1; *Orussus abietinus*: XP_012276889.1; *Polistes fuscatus* : XP_043495335.1; *Polistes annularis*: AAD52615.1; *Vespa velutina*: XP_047357816.1; *Vespula pensylvanica* : XP_043674637.1; *Ips typographus*: XP_081835128.1. (D) Venom serine protease precursor. *Apis mellifera* : XP_026296056.1; *Bombus impatiens* : XP_033177790.1; *Bombus terrestris* : XP_012163168.2; *Halyomorpha halys* : XP_066907599.1; *Megachile rotundata* : XP_003700816.2; *Polistes dominula*: XP_015184241.1; *Vespa crabro* : XM_046975037.1; *Vespa velutina*: XM_047505861.1; *Vespula vulgaris*: XM_051003336.1; *Vespula pensylvanica* : XM_043821360.1.

According to the SwissProt database, there were 842 proteases, among which 20 were proteases and 1294 proteins were nonenzymatic proteins, with 2 toxic proteins. Based on sequence similarity, the 22 toxic proteins were classified into four categories: 15 venom allergens, 2 dipeptidyl peptidases, 1 chemokine, and 5 esterases (Table 1; Figure 2). Moreover, the hyaluronidase (Maker000642), venom dipeptidyl peptidase 4 (Maker002446), and phospholipase A1 (Maker005828) were identified (Table S1; Figure 3). Phylogenetic analyses of the four genes (hyaluronidase, venom dipeptidyl peptidase 4, phospholipase A1, and venom serine protease precursor) showed that the venom genes of Vespidae clustered together in each of the phylogenetic tree. This indicated that the divergence of these genes was consistent with species divergence.

**TABLE 1:** Venom protein annotation of *V. mandarinia* via the SwissProt, iVenomDB, and T3DB databases.

Classification	Gene	Swiss‐Prot	iVenomDB	T3DB
Venom allergen	Maker005828	Phospholipase A1 2	Phospholipase A1	
	Maker006726	Phospholipase A2 group XV	Group XV phospholipase A2	
	Maker008236	Phospholipase A2	Acidic phospholipase A2 PA4‐like isoform X2	
	Maker000972	Venom acid phosphatase Acph‐1	Venom allergen acid phosphatase	
	Maker001601		Venom allergen 3‐like	
	Maker001603	Venom acid phosphatase Acph‐1	Venom allergen acid phosphatase	
	Maker003536		Venom allergen acid phosphatase	
	Maker006667	Venom allergen 5	Venom allergen 5	
	Maker000642	Hyaluronidase		
	Maker005535	Venom serine carboxypeptidase		
	Maker007620	Venom serine protease		
	Maker010541	Venom serine protease 34		
	Maker001770	Mitogen‐activated protein kinase kinase kinase 15		
	Maker002270	Mitogen‐activated protein kinase p38b		Mitogen‐activated protein kinase 11
	Maker005117	Mitogen‐activated protein kinase‐binding protein 1		
	Maker006962			Mitogen‐activated protein kinase 13
	Maker007926	Mitogen‐activated protein kinase 8		Mitogen‐activated protein kinase 10
	Maker007927	Mitogen‐activated protein kinase 1		Mitogen‐activated protein kinase 1
	Maker009674			Mitogen‐activated protein kinase 13
Dipeptidyl peptidase	Maker002446	Venom dipeptidyl peptidase 4	Venom dipeptidyl peptidase 4	Dipeptidyl peptidase 4
	Maker006850	Dipeptidyl peptidase 4	Venom dipeptidyl peptidase 4‐like isoform X2	Dipeptidyl peptidase 4
	Maker010594		Venom dipeptidyl peptidase 4	Dipeptidyl peptidase 4
Esterase	Maker001004	Esterase AGAP003155		
	Maker002060	Venom carboxylesterase‐6		
	Maker002119	Esterase FE4		Acetylcholinesterase
	Maker002120	Esterase FE4		Acetylcholinesterase
	Maker010427			Acetylcholinesterase
Chemokines	Maker000866	Layilin		
	Maker001131			Antithrombin‐III
	Maker004067			Antithrombin‐III
	Maker007739			Antithrombin‐III
	Maker002144			Coagulation factor IX
	Maker005093			Coagulation factor X
	Maker008000			Coagulation factor IX
	Maker011279			Coagulation factor VII

According to the annotations from iVenomDB, there were 11 toxicity‐related proteins, 7 thioredoxins, 2 calreticulin precursors, and 5 proteins related to serine proteases (Table S2). Eight toxic proteins shared with the SwissProt annotation were identified. Furthermore, the venom allergen acid phosphatase (Maker003536) and venom allergen 3‐like (Maker001601) were identified. In the T3DB database, 18 toxic proteins were identified. Two other chemokines, antithrombin‐III (Maker001131; Maker004067; Maker007739) and coagulation factors (Maker002144), which were not found in the SwissProt annotation, were identified (Table S3).

The GO‐BP analysis revealed that these proteins were related mainly to “neutrophil degranulation”, “translational initiation”, and “translation”. Moreover, there were nine pathways possibly related to the inflammatory pathway, which included “neutrophil degranulation”, “NIK/NF‐kappaB signaling”, and “Fc–epsilon receptor signaling pathway” (Table S4).

According to the KEGG enrichment results, “signal transduction” was the most enriched pathway, with the exception of “global and overview maps” (level 2) (Tables S5 and S6). The proteins enriched in the “proteasome” pathway at level 3 might be related to the toxicity of *V. mandarinia*.

### 3.2 Peptidome

On the basis of the Swiss‐Prot database annotation, the peptide fragments were mapped to 293 genes (Table S7). Moreover, 70 peptides are proteases, among which one peptide was identified as a phospholipase A2 isozyme (Maker008236) (Table 2). Among the 223 nonenzymatic peptides, two were identified as vespid chemotactic peptide (Maker004030) and the mastoparan‐like peptide (Maker007383).

**TABLE 2:** Peptide functional annotation of *V. mandarinia* via the Swiss‐Prot, iVenomDB, and T3DB databases.

GeneID	Swiss‐Prot	iVenomDB	T3DB
Maker004030	Vespid chemotactic peptide 5 h OS = Vespa magnifica OX = 202,807 PE = 1 SV = 1		
Maker007383	Mastoparan‐like peptide 12c OS = Vespa magnifica OX = 202,807 PE = 1 SV = 1	Mastoparan‐like peptide 12c	
Maker008236	Phospholipase A2 isozymes PA3A/PA3B/PA5 OS = *Heloderma suspectum* OX = 8554 PE = 1 SV = 3	Acidic phospholipase A2 PA4‐like isoform X2	
Maker011279			Coagulation factor VII
Maker008688			Mitogen‐activated protein kinase 14

In the iVenomDB database annotation, 7 peptides were identified, including 4 thioredoxin‐related proteins, one serine protease homolog, one mastoparan‐like peptide, and one acidic phospholipase A2 PA4‐like isoform X2 (Table S8).

Ninety peptides were identified according to the T3DB database; coagulation factor VII (Maker011279) and mitogen‐activated protein kinase 14 (Maker008688) were identified (Table S9).

According to enrichment analysis, 264 genes were enriched in GO‐BP terms. The GO‐BP analysis revealed few items possibly related to the inflammatory pathway, which included “neutrophil degranulation”, “cellular response to interleukin‐7”, “NIK/NF‐kappaB signaling”, and so on (Table S10).

According to KEGG enrichment analyses, 99 peptides were enriched. Moreover, “transport and catabolism” and “translation” were the most enriched pathways, with the exception of “global and overview maps” (Tables S11 and S12). The peptides were also enriched in the “proteasome” pathway.

### 3.3 Metabolome

The data from Compound Discoverer 3.1 revealed 2314 ion features, and 918 compounds were tentatively identified in positive mode. In negative mode, 1073 ion features were detected, and 499 compounds were identified (Tables S13 and S14).

According to the identified positive compounds, there were 218 gradable compounds and 700 non‐gradable compounds. The compounds were divided into 18 superclasses, and the most abundant superclass was organic heterocyclic compounds. Moreover, the most abundant class of organic heterocyclic compounds was indoles and their derivatives. In addition, dopamine, acetylcholine, and serotonin were identified.

According to the identified negative compounds, there were 132 gradable compounds and 367 nongradable compounds. The compounds were divided into 14 classes. Organic acids and derivatives constituted the most abundant classes of metabolites, primarily composed of carboxylic acids and their derivatives. γ‐Aminobutyric acid (GABA), N‐acetylhistamine, tryptamine, and dopamine are toxicity‐related compounds in negative mode.

## 4 Discussion

Vespids are among the most medically significant in Hymenoptera because of the high lethality of the venom. Venom widely exists in Metazoa and is involved in diverse biological activities; among these activities. Ants, wasps, and bees rely on venom as a tool for both predation and self‐defense (Schendel et al. 2019). Wasp venom was a complex mixture of enzymes, peptides, allergens, amino acids, amine substances, and other bioactive components (Konno et al. 2016; Nakajima 1986). According to a previous study, the venom of *A. mellifera* is primarily composed of “melittin (a 26‐residue, cysteine‐free amphipathic peptide), phospholipase A2 (PLA2), histamine, hyaluronidase, and several small peptides” (Hoffman 1987). The emerald cockroach wasp ( *Ampulex compressa* ) paralyzes its host ( *Periplaneta americana* ) using venom (Arvidson et al. 2019).

PLA1, hyaluronidase, DPPIV, and mastoparan‐like peptide have been experimentally confirmed as major allergens within the *Vespa* genus (Pretre et al. 2022; Sookrung et al. 2014; Ye et al. 2023). Additionally, PLA2, C‐type lectins, and hyaluronidase found in *V. mandarinia* have been identified as toxic components in snake venom. PLA2 was experimentally confirmed in *Micrurus lemniscatus* coral snake venom that could induce hemolysis, cytotoxicity, and neurotoxicity (Oliveira et al. 2008; Tang et al. 2023). C‐type lectins primarily cause cytotoxicity (Eble 2019; Torres et al. 2026). The glands of monocled cobra ( *Naja kaouthia* ) contained PLA2 and other components (Xu et al. 2017).

We identified PLA1, venom dipeptidyl peptidase 4, and venom serine protease precursor in *V. mandarinia* venom. PLA1 has been identified as the major lethal agent in *V. basalis* and *V. verutina* venom, exhibiting potent hemolytic and significant lethality in murine models (Ho et al. 1999; Ho and Ko 1988). Moreover, in *V. affinis* and *P. paulista* venom, PLA1 was also a key allergenic component (Santos et al. 2007; Sukprasert et al. 2013).

Hyaluronidase, which can hydrolyze hyaluronan in human skin, has been identified in hornets, yellow jackets, paper wasps, etc. (King and Wittkowski 2011). Thus, hyaluronidase, as a “spreading factor,” promotes the permeability of vessels and tissues to help other toxins diffuse quickly (Habermann 1972; Lee et al. 2016).

Venom dipeptidyl peptidase 4 was one of the minor components of *Vespula vulgaris* (Blank et al. 2010; Ollert and Blank 2015). In *Vespa basalis*, DPPIV matured to mastoparan B (MP‐B), which was its major toxin peptide and had hemolytic activity (Lee et al. 2007). DPPIV sequences show high similarity across the genera *Vespa* and *Vespula* (Monsalve et al. 2023). The study in *Vespa basalis* showed MP‐B was a pain‐inducing component in the venom (Zhou et al. 2020). γ‐Aminobutyric acid (GABA), N‐acetylhistamine, tryptamine, and dopamine were confirmed to have toxicity in spiders and wasps (Aroniadou‐Anderjaska et al. 2023; Endo 1979; Lee et al. 2025; Maus et al. 2002; Prajapati et al. 2024; Rádis‐Baptista and Konno 2024). They were also found in *V. mandarinia*. In general, these venom components are likely to contribute to the biological activity of *V. mandarinia* venom. The aggressive behavior of *V. mandarinia* was commonly attributed to its large size, flight capability, especially the venom (Baracchi et al. 2015; Kwon and Choi 2020; Nalepa and Swink 2018). Previous studies have suggested that the venom of *V. mandarinia* exhibits higher toxicity than closely related species, such as *Vespa basalis* and *Vespula vulgaris*. Except for the common “spreading factors” like hyaluronidase, the venom of *V. mandarinia* also contains phospholipase A1 (PLA1).

Our methodology does have certain limitations. Using venom sacs included portions of adjacent tissues unavoidably, which led to the identification of certain tissue‐derived proteins. Proteins with unknown functions or not contained in public databases cannot be identified through sequence similarity. In subsequent studies, isolation and function validation of the most biologically significant venom proteins need to be conducted.

## 5 Conclusions

These findings provided a comprehensive multi‐omics dataset of *V. mandarinia* venom, elucidating the molecular basis of its toxicity and biological functions. Subsequent research will prioritize the functional validation of pivotal toxic components to elucidate venom‐mediated effects.

## Author Contributions

**Yuan‐Chong Shi:** formal analysis (lead), investigation (equal), visualization (equal), writing – original draft (lead). **Hai‐Feng Mo:** investigation (supporting), writing – original draft (supporting). **Shu‐Hui Yu:** conceptualization (supporting), methodology (supporting), resources (lead), writing – review and editing (supporting). **Xiao‐Yun Niu:** investigation (supporting), writing – original draft (supporting). **Pu Yang:** conceptualization (lead), formal analysis (equal), funding acquisition (lead), investigation (equal), methodology (lead), supervision (lead), writing – review and editing (lead).

## Funding

This work was supported by Young‐ and Middle‐Aged Academic and Technical Leaders Reserve Talent Project of Yunnan Province, 202105AC160031. Chinese Academy of Forestry Outstanding Youth Support Program, CAFYBB2023QB007.

## Conflicts of Interest

The authors declare no conflicts of interest.

## Supporting information


**Table S1:** Protein identification details for *V. mandarinia*.

**Table S2:** iVenomDB annotation of proteins identifications of *V. mandarinia*.

**Table S3:** T3DB annotation of proteins identifications of *V. mandarinia*.

**Table S4:** GO enrichment analysis of *V. mandarinia* proteins.

**Table S5:** KEGG pathway enrichment analysis of *V. mandarinia* proteins.

**Table S6:** KEGG pathway analysis of *V. mandarinia* proteins.

**Table S7:** Peptide identification details for *V. mandarinia*.

**Table S8:** iVenomDB annotation for peptide identifications of *V. mandarinia*.

**Table S9:** T3DB annotation for peptide identifications of *V. mandarinia*.

**Table S10:** GO analysis of *V. mandarinia* peptides.

**Table S11:** KEGG pathway enrichment analysis of *V. mandarinia* peptides.

**Table S12:** KEGG pathway analysis of *V. mandarinia* peptides.

**Table S13:** List of positively identified metabolites of *V. mandarinia*.

**Table S14:** List of negative identified metabolites of *V. mandarinia*.

## Data Availability

The proteomic data was submitted to the PRIDE with accession number PXD056368. The metabolomic data was submitted to MetaboLights with accession number MTBLS11304.
